# Potential of Neuroinflammation-Modulating Strategies in Tuberculous Meningitis: Targeting Microglia

**DOI:** 10.14336/AD.2023.0311

**Published:** 2024-05-07

**Authors:** Huan-Jun Lu, Daji Guo, Qian-Qi Wei

**Affiliations:** ^1^Institute of Special Environmental Medicine, Nantong University, Jiangsu, China; ^2^Department of Neurology, Sun Yat-sen Memorial Hospital, Sun Yat-sen University, Guangzhou, China; ^3^Department of Infectious Diseases, General Hospital of Tibet Military Command, Xizang, China

**Keywords:** tuberculous meningitis, neuroinflammation, host-directed therapy, microglia, cytokines

## Abstract

Tuberculous meningitis (TBM) is the most severe complication of tuberculosis (TB) and is associated with high rates of disability and mortality. Mycobacterium tuberculosis (M. tb), the infectious agent of TB, disseminates from the respiratory epithelium, breaks through the blood-brain barrier, and establishes a primary infection in the meninges. Microglia are the core of the immune network in the central nervous system (CNS) and interact with glial cells and neurons to fight against harmful pathogens and maintain homeostasis in the brain through pleiotropic functions. However, M. tb directly infects microglia and resides in them as the primary host for bacillus infections. Largely, microglial activation slows disease progression. The non-productive inflammatory response that initiates the secretion of pro-inflammatory cytokines and chemokines may be neurotoxic and aggravate tissue injuries based on damages caused by M. tb. Host-directed therapy (HDT) is an emerging strategy for modulating host immune responses against diverse diseases. Recent studies have shown that HDT can control neuroinflammation in TBM and act as an adjunct therapy to antibiotic treatment. In this review, we discuss the diverse roles of microglia in TBM and potential host-directed TB therapies that target microglia to treat TBM. We also discuss the limitations of applying each HDT and suggest a course of action for the near future.

## Introduction

Tuberculosis (TB) is a major cause of death, resulting from a single infectious agent, Mycobacterium tuberculosis (M. tb). In 2022, the annual global TB report published by the World Health Organization (WHO) estimated that 10.6 million people developed TB disease globally and 1.4 million TB deaths occurred among human immunodeficiency virus (HIV)-negative people. Since 2019, the COVID-19 pandemic has detrimentally affected TB diagnosis and treatment, increased the TB burden, and slowed, halted, or reversed the battle against TB [1]. Moreover, TB is a contagious illness that often presents as lung disease (pulmonary TB) and affects other body regions (extrapulmonary TB). Tuberculous meningitis (TBM) is the most severe manifestation of TB, accounting for approximately 5% of extrapulmonary TB, and is characterized by inflamed meninges as the M. tb disseminates in the subarachnoid space [2, 3]. With current ineffectual therapies, TBM is devastating, debilitating, and associated with high-mortality and several neurological complications, including hydrocephalus, stroke, cranial nerve palsies, and epileptic seizures [4]. Much of the damage caused by TBM results from host-derived inflammatory responses, frequently due to non-productive neuroinflammation, in which microglia play a major initiating role [5]. Although microglia serve as immune supervisors in the central nervous system (CNS), their prolonged activation causes hyper neuroinflammation, which has the potential to be neurotoxic and impair the cognition of patients [6].

Host-directed therapy (HDT) is intended to be an adjuvant to traditional anti-tuberculous therapy (ATT) owing to its immunomodulatory effects and immune augmentation and has shown promising results in the treatment of TB in recent years [7-9]. Several excellent reviews on TBM and neuroinflammation have been reported [5, 9, 10], such as Spanos et al. has discussed the crucial immune regulatory role of microglia to TB in the CNS. However, there has been no review on the potential of HDT treatment on TBM [5]. In this review, we examined the current advances in microglial studies related to TBM and the application of HDT compounds to target the microglial pathway for treating TBM.

## Pathogenesis of TBM

TB infection commonly originates from the inhalation of aerosol particles containing M. tb [11]. After surviving the immune response of lung alveolar macrophages, neutrophils, and dendritic cells, M. tb establishes a primary infection in the lung parenchyma with typical granulomatous inflammation [12]. Depending on the patient’s immune status and the virulence of the bacillus, the M. tb infection may be halted by the formation of granulomas, in which macrophages, epithelioid cells, and lymphocytes gather to establish a latent infection, or it may disseminate from local invasion to the blood and lymphatic systems, which is common in both miliary and extrapulmonary TB [4, 13, 14]. Various mechanisms underlie the hematogenous translocation of M. tb from the respiratory epithelium to the meninges. M. tb directly invades the endothelial cells, and the subsequent dissemination is exacerbated by the presence of intracellular survival and persistence in myeloid cells, including macrophages, neutrophils, monocytes, and dendritic cells (as a metaphor for “trojan horse”), which escapes from phagocytosis and promotes virulence factor for migration [15-21]. Moreover, the blood-brain barrier (BBB) forms a strict boundary between the circulating blood and neural tissues. Without endocytic vesicles and fenestration, the specialized brain microvascular endothelial cells encircle the capillaries via intercellular tight junctions, interacting concertedly with multiple cell types like pericytes, microglia, astrocytes, and neurons to maintain CNS homeostasis, especially to guard against pathogen-caused damage [22, 23]. According to the detailed hypothesis by Rich and McCordock in 1933, small TB foci forms in the leptomeninges, subpial cortex, and subependymal region after hematogenous dissemination. This hypothesis is known as “Rich focus” rupture leads to the release of the bacilli into the subarachnoid space or parenchyma, inducing a single meningeal or para-meningeal granuloma, which is usually observed after autopsy [10, 11]. Additionally, they insisted that miliary TB and TBM have distinct identities because neither condition was observed together. However, Donald *et al*. in 2005 claimed that miliary TB was closely associated with the development of TBM, particularly in young children, because apparent bacillemia contributed to the establishment of a rich focus in the meninges or subcortical areas [24]. Moreover, another study demonstrated that superficial intraparenchymal granulomatous inflammation is a transpiral or perivascular extension (Virchow-Robin spaces) of leptomeningeal granuloma [25]. Without the support function of the astrocytic foot, the BBB is susceptible to TB infection in subarachnoid blood vessels, where the physiological structure allows bacilli to multiply and a biofilm-like community to develop.

When inflammation occurs in the meninges, a “dense and tenacious exudate” accumulates primarily in the basal cistern [4, 26]. The most common cause of elevated intracranial pressure, which consequently leads to hydrocephalus in patients with TBM, is the blockage of the basal cistern, absorptive arachnoid granulations, or physical obstruction of the tuberculoma at the cerebral aqueduct or fourth ventricle outlet [27]. Exudates containing erythrocytes, neutrophils, macrophages, and lymphocytes cause inflammation and necrosis, contributing to blood vessel pathology [28]. Moreover, the middle cerebral artery and its perforating branches to the internal capsule and basal ganglia are commonly affected [29].

**Figure 1.**
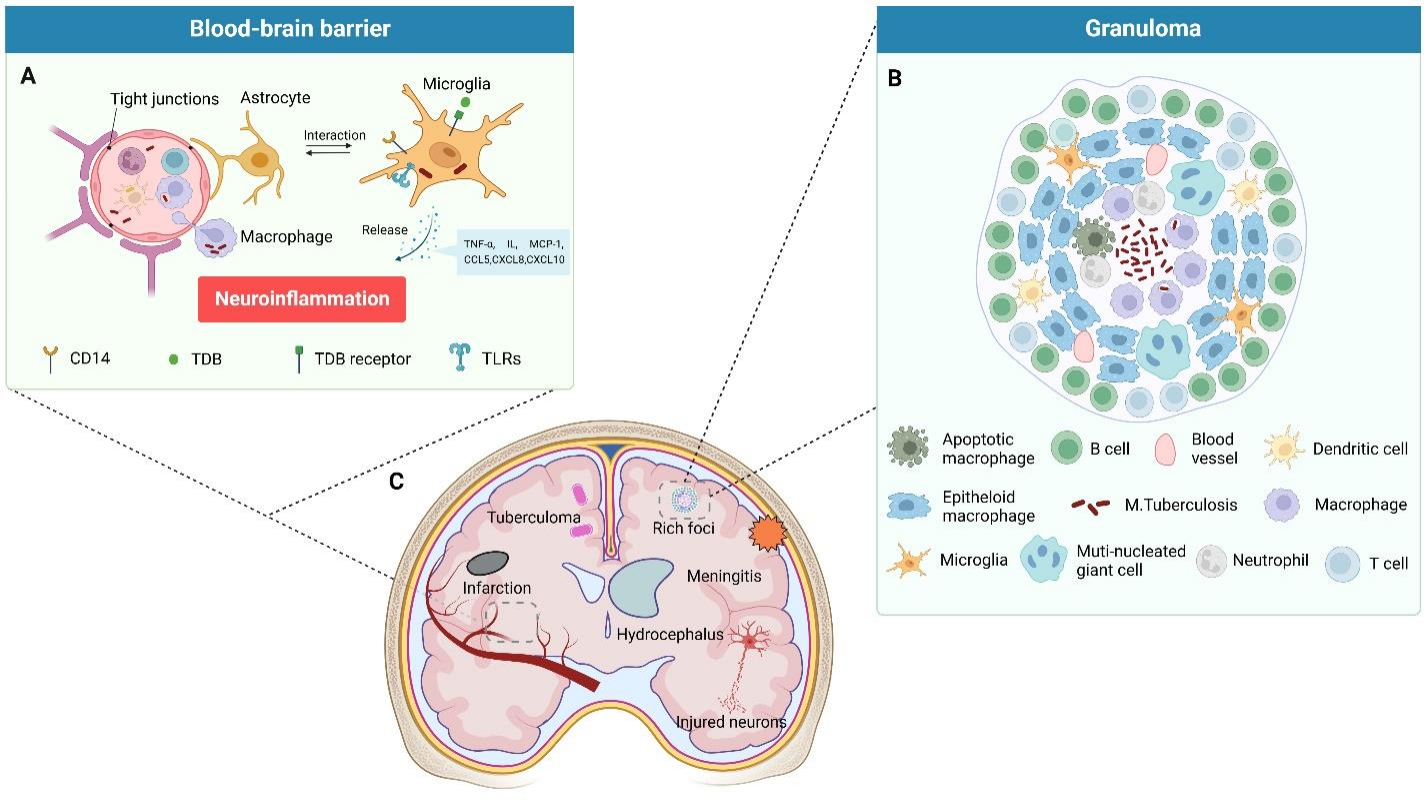
**The formation of granuloma in TBM and its common associated complications**. (**A**) The “trojan horse” model indicates that M. tb resides in macrophages, dendritic cells, and neutrophils, disseminating with blood infection usually following with establishment of miliary TB. Microglia become the main target of M. tb under the participation of TLRs in the brain after infected cells translocate across the BBB. Interacting with astrocytes, activated microglia release a wide range of cytokines and chemokines, which evokes the immune response, increasing the risk of hyperinflammation. (**B**) Schematic diagram of granuloma in the central nervous system. The ruptures of these small granulomas, also termed as “Rich foci,” are regarded as critical events in the development of TBM. C. Common complications induced by M. tb infection in the brain. Hyperinflammation and neuron injury are the common causes of death or disability. The figure was created with BioRender.com.

A retrospective study showed that 144 of 559 (25.8%) patients admitted with TBM had cerebral infarcts diagnosed with brain imaging, with three-quarters of them being acute or subacute [30]. In addition, hyponatremia and tuberculous brain abscesses are common macroscopic manifestations of TBM, and cognitive impairment occurs in approximately half of the patients with TBM concurrently infected with HIV, which cannot be ascribed to limited focal structural deficits such as tuberculomas [6]. These characteristics can exist separately or in combination and may not be detected radiographically until the disease has progressed (Fig. 1).

## Biological Roles of Microglia in TBM

Microglia are resident macrophages of the CNS and are historically believed to originate from the neuroectoderm, where other glial cells and neurons are established [31]. Recent studies have suggested that microglia originate from the yolk sac, which belongs to the mononuclear phagocytic system, and colonize the developing CNS before vasculogenesis [32, 33]. In contrast to most mature macrophages, microglia are maintained throughout life independent of blood input, with a self-renewal capacity to maintain balanced apoptosis and proliferation under physiological conditions [32, 34, 35]. Microglia are defined as either “resting” or “activated” in traditional notion. Numerous factors, including severe mechanical brain trauma or peripheral nerve injury caused by viral infections, may induce “resting microglia” to proliferate and change their immunophenotype, activating an inflammatory response [36]. The idea of lethargic “resting microglia” has been contested in recent studies. Although their somata are stationary, “resting microglia” continuously explore their surroundings, which can encompass an area larger than 10-fold their body and engage in intense interactions with nearby cells [37]. In the healthy brain, they appear to exist as a broad spectrum of distinct but overlapping phenotypes with diverse functions, including debris removal, neuron nourishment, pathogen protection, and homeostatic functions [38-40].

Although neurons and astrocytes are potential targets for M. tb, microglia are the primary targets in CNS because they share many, if not all, properties with macrophages [41, 42]. M. tb efficiently infects microglia through CD14 receptors and grows inside, like in macrophages [43, 44]. After exposure to M. tb H37Rv strain for 24h *in vitro*, human microglia and astrocytes exhibit intracellular infections. However, 76% of microglia are infected with M. tb containing an average of 4.2 bacilli/cell located within phagosomes, while 15% of astrocytes are infected with an average of 1.3 bacilli/cell [41].

Microglial cell identification by M. tb is through innate immune and neuro-specific receptors, particularly pattern recognition receptors (PRRs), which are required to successfully launch a fast response, even against the limited infiltration of T cells in the brain [45, 46]. Toll-like receptors (TLRs) are a form of PRR along with intracellular PRRs, C-type lectin receptors, complement receptor 3, triggering receptors expressed on myeloid cells (TREM), and myeloid DAP-12 associated lectin (MDL-1) [47]. Microglia express TLR1-TLR4, TLR5-TLR9, and the co-receptor CD14. Moreover, TLR1, TLR2, and TLR4 are expressed on the cell surface of microglia, whereas TLR3, TLR7, and TLR8 are expressed intracellularly (i.e., in endosomes) [47-49]. The role of TLRs in neurodegenerative disorders (such as Alzheimer’s disease (AD) and Parkinson’s disease (PD)), pathogenic infections (Neisseria meningitides, Neisseria meningitides, Japanese encephalitis virus (JEV), West Nile virus, and HIV), and ischemic brain injury has been studied extensively [50-56]; however, their function during TBM remains unexplored. Studies have shown that TLR4 binds to CD14 through lipopolysaccharides and participates in the internalization of M. tb[43]. In contrast, host macrophages internalize M. tb via TLR2, TLR4, and TLR9 pathways, activating downstream phosphorylation and cytokine production [57-59]. Similar to macrophages and their effective usage of PRRs in pathogen or damage recognition, understanding the underlying mechanisms of PRRs produced by microglia during TBM development is crucial. An old previous study showed that neither TLR2 nor dectin-1 is involved in sonicated M. tb-induced inflammatory responses in murine microglia, indicating that there is the participation of an undiscovered pathogen recognition mechanism during the identification of the M. tb antigen [60]. Interestingly, a recent study showed a potentially unidentified receptor of microglia for binding to a macrophage-inducible C-type lectin agonist (Mincle, a PRR allocated to the C-type lectin receptor family) [61]. A novel adjuvant of the TB subunit vaccine, trehalose-6,6-dibehenate (TDB), is a synthetic analog of trehalose-6,6-dimycolate (TDM, also known as mycobacterial cord factor) that is currently being tested in human clinical studies. Similar to TDM, TDB has the affinity for binding to Mincle and inducing spleen tyrosine kinase (Syk)-dependent signaling cascades for inflammatory gene expression among macrophages and dendritic cells [62, 63]. Despite the limited expression of Mincle in cultured primary microglia, TDB attenuates TLR4-mediated neuroinflammation in Mincle-knockout microglial cells and mice in a Syk-independent manner, which has conflicting effects compared to its role in macrophages. The receptor that recognizes TDB and triggers the phospholipase C-gamma 1 (PLC-γ1) signaling pathway remains unknown, necessitating further studies to solve this puzzle.

**Table 1:** Confirmed upregulated expression of molecules in microglia activated by LPS and *M. tuberculosis.*

Maker	Type	Description	LPS	M. tb	reference
**TNF-α**	Cytokine	Pro-inflammatory cytokine; Classical M1 stimuli;	Yes	Yes	[41, 44, 60, 64, 68, 70, 231, 232]
**IFNγ**	Cytokine	Pro-inflammatory cytokine; Anti-microbial functions; Initiating M1 phenotype	Yes	No	[64, 232, 233]
**IL-1α**	Cytokine	Pro-inflammatory cytokine	Yes	No	[44, 232]
**IL-1β**	Cytokine	Pro-inflammatory cytokine; Classical M1 stimuli;	Yes	Yes	[41, 64, 68, 70, 72, 231, 232]
**IL-2**	Cytokine	Pro-inflammatory cytokine	Yes	No	[64, 231, 232]
**IL-6**	Cytokine	Pro-inflammatory cytokine	Yes	Yes	[41, 60, 68-70, 231, 232]
**IL-10**	Cytokine	Immunomodulatory cytokine	No^#^	No	[44, 154, 234]
**IL-12**	Cytokine	Regulates natural killer cells response and the differentiation to Th1 cells	Yes	Yes	[60, 232, 235, 236]
**IL-18**	Cytokine	Pro-inflammatory cytokine; Belongs to the IL-1 family	Yes	Yes	[72, 237, 238]
**G-CSF**	Cytokine	Promotes neuronal survival, proliferation, differentiation, mobilization of hematopoietic stem and progenitor cells	Yes	Yes	[64, 232, 239]
**GM-CSF**	Cytokine	Induces hematopoiesis; drive and regulate inflammatory responses; inhibit antitumor immunity	Yes	Yes	[64, 232, 240]
**CCL2**	Chemokine	Recruits monocytes into sites of inflammatory responses and tumors	Yes	Yes	[41, 68, 69, 231, 241]
**CCL4**	Chemokine	Induces chemotaxis and adhesion of immune cells	Yes	Yes	[69, 242, 243]
**CCL5**	Chemokine	Activates NF-*κ*B pathways; recruits T lymphocytes and NK cells, participates in angiogenesis of tumor	Yes	Yes	[41, 232, 244]
**CXCL8**	Chemokine	Interacts with CXCR1 and CXCR2, stimulus for recruitment of MDSCs to tumor niche	Yes	Yes	[41, 245, 246]
**CXCL10**	Chemokine	Promotes the chemotactic activity of CXCR3+ cells; contributes to inflammatory pain and neuropathic pain;	Yes	Yes	[41, 68, 246, 247]
**iNOS**	Metabolic enzyme	Oxidative damage	Yes	Yes	[68, 69, 248]

# There are controversial results about IL-10 levels in the experiments with LPS-stimulated BV2 microglia.

After internalization by microglia, M. tb bacilli persist and multiply within the cells [44, 64]. For TBM, microglia are recruited and activated in the TB lesion region and are involved in granulomatous formation [25, 65]. Foamy macrophages exist in the center of CNS granuloma with varying degrees of necrosis and calcification and are surrounded by microglia, lymphocytes, epithelioid histiocytes, and new blood vessels [25, 65]. M. tb has developed strategies to survive inside macrophages by disrupting phagosome-lysosome fusion and preventing normal host trafficking events [66, 67]. However, to demonstrate the unique immuno-modulatory niche throughout the early and developing phases of TBM, more specific information regarding the parallel course of M. tb replication in microglia is required. In addition to direct stimulation by invading M. tb, the intricate network of interactions between migrating activated macrophages and microglia controls the active production of a wide range of cytokines, chemokines, and pro-inflammatory immunological markers in microglia. Moreover, it influences the variation of tumor necrosis factor-α (TNF-α), interleukins (IL), IL-1α, IL-1β, IL-6, IL-10, IL-12p40, IL-18, chemokines C-C chemokine ligand 2 (MCP-1/CCL2), CCL5, C-X-C motif chemokine ligand 8 (CXCL8), CXCL10, matrix metallopeptidase-1 (MMP-1), MMP-3, MMP-9, macrophage inflammatory protein-1β (MIP-1β), granulocyte colony-stimulating factor (G-CSF), and granulocyte-macrophage colony-stimulating factor (GM-CSF) [41, 44, 60, 64, 68-72] (Table 1).

The different roles of these abovementioned secretions have been discussed in previous reviews [5]. Under most circumstances, human hosts restrict M. tb infection to a state of silencing via a regulated homeostatic immune response. One of the reasons for death is the harm caused by bacterial invasion and virulence, for which antibiotic therapy with adequate penetration across the BBB and efficacy remains insufficient. Additionally, non-productive immune responses result from the excessive activation of microglia, triggering excessive cytokine secretion and inducing tissue damage in the CNS, where infarction, exudation, and ischemic injury induced by neuroinflammation have become the leading causes of severe cerebral accidents. In conclusion, M. tb directly damages microglia, neurons, and astrocytes through antigen recognition. However, the immune response of the host ultimately determines the nature of the disease [9]. Consequently, HDT may be a promising approach to restore the equilibrium of the inflammatory response and improve the germicidal effects of antibiotics as an adjunct therapy in treating TBM by targeting microglia. The following sections highlight several prospective HDTs and discuss their current utilization (Table 2).

**Table 2:** Different classes of HDT drugs that are promising for the treatment of TBM.

Classification	Compounds	Rationale for use in TBM	Clinical trials for TB/TBM (ClinicalTrials.gov)
**Corticosteroids**	Dexamethasone Prednisolone	Reduce mortality of TBM; Control inflammation and cerebral edema; Modulate cytokines and MMPs; Restrict PR	Leukotriene A4 Hydrolase Stratified Trial of Adjunctive Corticosteroids for HIV-uninfected Adults with Tuberculous Meningitis (NCT03100786) High-dose Rifampicin for the Treatment of Tuberculous Meningitis: A Dose-finding Study (NCT02169882) Study Of the Long-Term Outcome of Tuberculous Meningitis in Vietnamese Adults Treated with Adjunctive Dexamethasone (NCT01317654) The Relationships Between Gene Polymorphisms of LTA4H and Dexamethasone Treatment for Tuberculous Meningitis (NCT02588196) Adjunctive Corticosteroids for Tuberculous Meningitis in HIV-infected Adults (NCT03092817)
**Glutaminase inhibitors**	DON JHU083 BPTES CB-839	Increased glutaminase induces microglial phenotype shifting; Reverse the expression of TNF-α and iNOS;	/
**MMP inhibitors**	Doxycycline Marimastat Batimasat Cipemastat Minocycline	Inhibit microglial pro-inflammatory cytokines; Reduces collagenase activity; Restore dysregulated gene expression patterns; Improve the cavitation; Marimastat has a good penetration of BBB; Reduce granuloma formation and bacterial load; Consolidate granuloma architecture and reduce blood vessel leakage	Doxycycline in Human Pulmonary Tuberculosis (NCT02774993) Utilizing the Crosstalk Among Chicoric Acid, 13-Cis Retinoic Acid (Aerosolized), Minocycline and Vitamin D as a Potent Quadrate Therapy for Treating Patients with Multidrug-resistant TB and Patient with Both Multidrug-resistant TB and COVID-19 (NCT05077813)
**NSAIDs**	Aspirin Ibuprofen	Inhibit tuberculosis infection in mice models; Reduce brain infarction and related mortality; Mediate cytokines without impairing primary microglia	A Pilot Study of Adjunctive Aspirin for the Treatment of HIV Negative Adults with Tuberculous Meningitis (NCT02237365) Intensified Tuberculosis Treatment to Reduce the Mortality of Patients with Tuberculous Meningitis (NCT04145258) Linezolid, Aspirin and Enhanced Dose Rifampicin in HIV-TBM (NCT03927313) Potential Efficacy and Safety of Using Adjunctive Ibuprofen for XDR-TB Tuberculosis (NCT02781909) Adjunctive Acetylsalicylic Acid and Ibuprofen for Tuberculosis (NCT04575519)
**PDE inhibitors**	Cilostazol Sildenafil Roflumilast Cilomilast Rolipram CC-11050	Reverse the expression of inflammatory genes in activated microglia; Improve bacterial burden with better clinical outcomes in animal models; Sustain BBB, prevent brain edema	TB Host Directed Therapy (NCT02968927)
**Statins**	Atorvastatin Simvastatin Rosuvastatin Pravastatin Fluvastatin	Affect secretion of microglia; Modulate phagosome maturation and autophagy; Interrupt cellular cholesterol-metabolism	Statin Adjunctive Therapy for TB: A Phase 2b Dose-finding Study of Pravastatin in Adults with Tuberculosis (NCT03882177) Statin Adjunctive Therapy for TB (NCT03456102) Treating Tuberculosis with the Lipid Lowering Drug Atorvastatin in Nigeria (NCT04721795) Atorvastatin to Reduce Inflammation After Tuberculosis Treatment Completion (NCT04147286)
**TNF-α Antagonists**	Etanercept Infliximab Adalimumab Thalidomide	Antagonize excessive TNF-α induced neuroinflammation; Decrease M. tb burden; Alleviate PR after ATT; Complement corticosteroids;	Placebo-Controlled Trial of Safety and Efficacy of Thalidomide in Patients with Infections Due to Mycobacterium and/or HIV (NCT00002104) Phase II Placebo-Controlled Study of Thalidomide in Patients with Mycobacterial and HIV Infections (NCT00004276)
**Vitamin D**	Cholecalciferol	Deficiency in vitamin D impairs immunity against M. tb; Promote anti-inflammatory state in microglia; Guarantee phagosome maturation Participate in the innate immune process	Impact of Vitamin D Supplementation on Host Immunity to Mycobacterium Tuberculosis and Response to Treatment (NCT00918086) Effect of Vitamin D as Adjunctive Therapy in Patients with Pulmonary Evolution Tuberculosis (NCT02464683) Vitamin D Supplementation Effect in Children with Pulmonary Tuberculosis Treatment (NCT05073965) Vitamin D to Resolve Inflammation After Tuberculosis (NCT03011580)

ATT, anti-tuberculous therapy; BBB, blood-brain barrier; HDT, host-directed therapy; HIV, immunodeficiency virus; iNOS, inducible nitric oxide synthase; MMPs, matrix metalloproteinases; M. tb, Mycobacterium tuberculosis; NSAIDs, non-steroidal anti-inflammatory drugs; PDE, Phosphodiesterase; PR, paradoxical reaction; TB, tuberculosis; TBM, Tuberculous meningitis; TNF-α, tumor necrosis factor-α.

## Corticosteroids

Corticosteroids have been regarded as a standard adjuvant to ATT since it was first used to reduce TBM inflammation in the early 1950s [73, 74]. Several clinical trials have evaluated the efficacy of corticosteroids (Table 2). A 2016 Cochrane systematic review of 1337 participants (with 469 deaths) diagnosed with TBM from nine trials concluded that corticosteroids in combination with ATT reduced deaths by nearly one quarter (risk ratio = 0.75, 95% confidence interval = 0.65 to 0.87; high-quality evidence) in the short term; however, no difference was found in the rate of disabling neurological deficits [75]. However, in a long-term follow-up study, dexamethasone did not improve the 5-year mortality rate in patients with severe TBM [76]. Corticosteroids reduce meningeal inflammation and cerebral edema, as well as restore vascular permeability [4, 75]. In a randomized clinical trial, adjuvant dexamethasone reduced cerebrospinal fluid (CSF) MMP-9 concentrations in HIV-negative adults with TBM [77]. Production of TNF-α, IL-6, IL-1β, CCL5, and CXCL10 by dexamethasone-treated microglia eliminated M. tb infection in a concentration-dependent manner after 24 hours [41]. These studies suggest that dexamethasone “impairs” microglia function. Moreover, dexamethasone inhibits phagocytosis, cell proliferation, and pro- and anti-inflammatory cytokine production in microglia, including IL-10, which is also observed in lipopolysaccharide (LPS)-activated microglial cells treated with dexamethasone [78, 79]. Nevertheless, long-term or high doses of corticosteroids disrupt phagocytosis of microglia, causing an abnormal immune stasis in CNS and making it difficult to eliminate foreign antigens or misfolded proteins like amyloid-β [80].

Another concern about corticosteroid usage is the paradoxical reaction (PR), identified by the worsening preexisting or appearance of new tuberculous lesions in patients whose clinical symptoms initially improve with ATT [81]. The proportion of HIV-negative patients with TBM who develop PR ranges from 31.2% to 56% [82-84]. After receiving standard ATT with adequate corticosteroids, patients usually experience worsening basal cistern exudates and hydrocephalus, frequently involving the cranial nerves [81, 85, 86]. Corticosteroid usage alone is insufficient in those PR cases, and whether corticosteroids reduce the morbidity of PR remains controversial [87]. Treatment regimens for TBM that depend on corticosteroids are inadequate, and further studies are required on other treatment options.

## Glutaminase inhibitors

Glutamate is the most abundant excitatory amino acid neurotransmitter released by neurons into the synaptic cleft in the CNS. It interacts with metabotropic receptors, including metabotropic glutamate receptors (mGluR), and ionotropic receptors, like N-methyl-D-aspartate receptor (NMDAR), to transmit excitatory signals [88, 89]. Glutamate uptake by astrocytes through the glutamate transporters, excitatory amino acid transporter (EAAT), is a sodium gradient. In astrocytes, glutamate is converted to glutamine-by-glutamine synthetase, released from astrocytes to regenerate glutamate in neurons. The process of recycling glutamate is the well-described “glutamate-glutamine cycle” [90, 91]. Excessive glutamate accumulation caused by the disruption of the glutamate-glutamine cycle or impaired BBB integrity is toxic and can trigger apoptosis in neurons owing to Ca^2+^ overload caused by the overstimulation of glutamate receptors [92, 93]. Moreover, neuronal death and glutamate spillover from the synaptic area can cause neuroinflammation[94].

Global transcriptome sequencing of ventricular CSF from pediatric patients treated for TBM revealed active pathways for glutamate neurotransmitter release. The binding and uptake of glutamate by NMDRA compared with other infection controls, such as bacterial meningitis, indicated that neuronal excitotoxicity was prominent in the ventricular CSF during TBM infection [95]. This process may be associated with an abundance of monocytes in the ventricles. Contrary to popular belief, cytokine signaling, and toll-like receptor pathways were less enriched in TBM compared with other infection controls. These findings suggest that glutamate-induced neuronal excitotoxicity may be more critical than previously thought. Similarly, using ^1^H NMR metabolomics, a research team confirmed a disrupted glutamate-glutamine cycle in TBM CSF samples from the South African population [96].

Both astrocytes and microglia are involved in glutamate modulation. However, microglia do not express glutamate transporters in the ‘resting’ state. Once reactivated, microglia begin synthesizing EAATs and glutamine synthetase to compensate for the impaired clearance of astroglial glutamate under pathological conditions [97-99]. In addition, glutaminase production by activated microglia, which catalyzes the conversion of glutamine to glutamate, is abnormally elevated in individuals with chronic CNS disorders, including AD, HIV-1-associated neurocognitive disorder (HAND), and acute brain injury [100-102]. Several studies on primary microglia and HIV-1 infected, JEV-infected, or LPS-treated microglia have demonstrated that excessive glutamate release directly correlates with upregulated glutaminase production, which is involved in the TNF-α pathway [101-103]. Moreover, increased glutaminase levels may activate microglia and change their cellular phenotype to a pro-inflammatory state [102]. These findings imply that aberrant pro-inflammatory cytokines of multiple origins (including those in an autocrine manner) can activate microglia and elevate glutaminase expression in the TBM. Glutamate overflow from a disrupted glutamate-glutamine cycle and glutamate released by microglia might amplify neural excitotoxicity and thus exaggerate neuroinflammation [104-106]. Currently, there are only a few studies on the misexpression of neurotransmitters and the related mechanisms involving microglia in TBM.

Considering that TBM shares similarities with HAND and ischemic stroke—the former also involves pathogen invasion and neuroinflammation, while the latter is a common complication of TBM—the findings of previous studies indicating that the inhibition of glutaminase is beneficial in these two disease models suggests a potential pathway for treating TBM (Table 2) [4, 107-109]. Notably, 6-diazo-5-oxo-L-norleucine (DON), a glutamine antagonist with a structure similar to glutamine, efficiently reverses cognitive decline in rodent HAND models by inhibiting glutamate synthesis through interacting with glutaminase in microglia [110]. Because the clinical application of DON is impeded due to its gastrointestinal toxicity, a more tolerant DON prodrug, JHU083, with enhanced brain delivery, has been tested in EcoHIV-infected mice [111]. It works similarly to DON, which decreases CSF glutamate concentration and glutaminase activity in microglia-enriched CD11b+ cells.

Bis-2-(5-phenylacetamido-1,2,4-thiadiazol-2-yl) ethyl sulfide 3 (BPTES) is another nonspecific but allosteric glutaminase inhibitor. Apart from DON, BPTES shares no commonalities with glutamine or glutamate; it silences glutaminase in a non-cooperative manner, where it interacts with other enzymes and receptors that detect glutamate or glutamine as substrates. Therefore, in the same dosage, BPTES may be less toxic than DON [112, 113]. Moreover, BPTES effectively abolishes LPS-induced microglial activation by reversing TNF-α and inducible nitric oxide synthase (iNOS) expression [102]. However, BPTES is poorly soluble in aqueous solvents and has a low glutaminase activity efficiency (IC50, 3μM). Therefore, further studies are required to identify a comparable substitute for BPTES as a pharmacological probe. Among the BPTES analogs, CB-839 is a potent oral glutaminase inhibitor with antiproliferative activity against multiple malignancies *in vitro* and *in vivo* [114-116]; it not only interrupts the metabolism of glutamine-dependent cancer cells but also improves immune cell activity in the tumor microenvironment [115, 116]. A previous study showed that CB-839 reverses ischemia-induced microglial activation, inflammatory responses, and exosome release in cerebral ischemic rat models [100]. Moreover, CB-839 is currently being tested for its effectiveness and toxicity in four phase II clinical studies, either alone or combined with antitumor medicines (clinicaltrials.gov). However, further clinical studies are required to confirm its role as a modulator of neuroinflammation.

## MMP inhibitors

Matrix metalloproteinases (MMPs), which comprise a family with 23 members in humans, are characterized by zinc-binding motifs [117]. MMPs are proteases that degrade extracellular matrix (ECM) components, including collagen and proteoglycans. Furthermore, they are vital in controlling the extracellular pools of inflammatory cytokines and chemokines, including TNF-α, IL-1β, CCL7, and CXCL5 [118, 119]. The widely discussed MMP-2 and MMP-9 are directly implicated in neuroinflammation, ischemia, and infarction in brain tissues and BBB permeation during several patho-physiological processes [120]. Microglia are the primary source of MMP-2 and MMP-9 in the CNS [121]. The mRNA expression levels of MMP-1, -3, -8, and -9 significantly increased in microglia treated with LPS or phorbol myristate acetate (PMA). Inhibition of MMP-3 and -9 significantly suppresses proinflammatory cytokines, indicating that they function as inflammatory mediators in microglia [122]. Evidence suggests that MMP-2 and MMP-9 levels are increased in the CSF of patients with TBM and correlate with disease severity and neurological complications [123-125]. MMP-9 has also been detected in granulomas in human brain biopsies and has been linked to BBB breakdown [126]. Although MMPs secretion in the CNS appears to be triggered due to a network initiated by M. tb-infected monocytes, microglia contain a wide range of MMP products independent of monocytes. In an *in vitro* study, the expression of MMP-1, -3, and -9 increased in human CHME3 microglial cells infected with M. tb, whereas the fold increase was significantly higher when stimulated by conditioned medium from M. tb-infected human monocytes (CoMTb) [71]. Despite the increase in other MMP transcripts, astrocytes have been observed to secrete MMP-9 in a monocyte-dependent manner under the same conditions [127]. Interestingly, CoMTb unexpectedly downregulated constitutive microglial MMP-2 gene expression, which is resistant to dexamethasone, given that MMP-2 and -9 share conserved sequences and are major substrates [119]. Therefore, microglia play a critical role in the MMPs network in response to M. tb stimuli [71].

Doxycycline, a chemically modified tetracycline antibiotic with broad-spectrum MMP-inhibitory activity, is the only MMP inhibitor licensed by the US Food and Drug Administration (FDA) (Table 2) [128]. It inhibits MMP-1 and MMP-3 secretion in M. tb-infected primary human macrophages and reduces collagenase activity owing to M. tb-induced MMPs [129-131]. A phase 2 double-blind clinical trial that recruited 30 patients with pulmonary TB was performed to evaluate the outcomes of doxycycline as an adjunctive treatment with standard antibiotic therapy [132]; 2 weeks of adjunctive doxycycline accelerated the normalization of dys-regulated gene expression patterns in patients with TB towards normal and downregulated type Ⅰ/Ⅱ interferon, which diminished in healthy individuals following effective anti-TB treatment. Doxycycline reduced MMP-9 expression in the blood transcriptome and had a long-term suppressive effect on plasma MMP-1 levels. In sputum samples of TB patients, MMP-1, -8, and -9 levels were decreased with impaired collagenase and elastase activities compared with those in placebo samples. Additionally, the radiographic results demonstrated that doxycycline enhanced the average volume of the pulmonary cavities on day 56, suggesting that the inhibition of MMPs improved the cavitation caused by M. tb. Furthermore, doxycycline has pleiotropic effects on the MMP family, assisting in treating pulmonary TB with long-term effectiveness, good tolerance, and a wide range of safety. However, its low penetration into brain tissues restricts its application in TBM treatment [133, 134].

Marimastat is another broad-spectrum metallo-proteinase inhibitor that has the potential to cross the BBB and block metalloproteinase activity in the brain [135]. In a lung tissue model of TB based on human lung-derived cells and primary human monocyte-derived macrophages, Parasa et al. found that marimastat reduced granuloma formation and bacterial load [136]. Treatment of M. tb-infected mice with only marimastat had little effect on the bacterial burden but markedly enhanced bacterial death compared with the frontline TB drugs, isoniazid (INH) and rifampicin (RIF), by approximately 10-fold. Additionally, marimastat helps consolidate the granuloma architecture and reduces blood vessel leakage [137]. As deformation and exudation of granulomas in the brain could be lethal in patients with TBM, it is reasonable to consider multiple specific MMP inhibitors, including marimastat, batimasat, and cipemastat, or other specific MMPs antibodies, such as an anti-MMP-9 monoclonal antibody, as potential adjunctive therapies [138, 139].

## NSAIDs

Non-steroidal anti-inflammatory drugs (NSAIDs) are classified based on their selectivity for inhibiting cyclooxygenase/prostaglandin-endoperoxide synthase (PGHS) enzymes, thereby interrupting the formation of pro-inflammatory and immunosuppressive mediators such as prostaglandins and leukotrienes, which are primarily used to treat pain and inflammatory conditions (Table 2) [7, 140, 141]. Aspirin and ibuprofen act synergistically with the frontline anti-TB drug pyrazinamide to inhibit TB infection in mouse tissues [142]. A recent randomized controlled clinical trial in HIV-negative adults with TBM who received dexamethasone and aspirin (81 or 1000 mg) or placebo daily as adjunctive therapy to ATT and dexamethasone reported that the aspirin group had a lower risk of new MRI-proven brain infarction and related deaths; however, these differences were not statistically significant. Moreover, CSF analysis revealed dose-dependent inhibition of thromboxane A2 and upregulation of pro-resolution protectins in the aspirin group [143]. Similar findings were obtained in a previous study, which found that aspirin reduced short-term stroke and mortality rates [144]. However, another trial in children with TBM demonstrated that aspirin did not significantly improve morbidity or mortality [145]. According to meta-analyses, aspirin reduced the risk of new infarctions or new-onset stroke in patients with TBM but did not significantly reduce mortality [146, 147]. Consequently, more extensive clinical trials are required to confirm the efficacy of aspirin in the treatment of TBM. Aspirin inhibits phagocytosis in LPS-challenged microglia and reduces the pro-inflammatory cytokines TNF-α and IL-1β levels while elevating the anti-inflammatory IL-10. Unlike dexamethasone, which influences the function of primary microglial cells, aspirin inhibits LPS-challenged microglia precisely without affecting unchallenged microglia [78, 148]. There is evidence that NSAIDs, including aspirin, have neuroprotective functions, and more attention should be paid to the relationship between NSAIDs and microglia in TBM [140, 149, 150].

## PDE inhibitors

Phosphodiesterases (PDE) are classified into 11 subfamilies (PDE1-11) with different substrate specificities; their primary targets are cyclic adenosine monophosphate (cAMP) and cyclic guanosine monophosphate (cGMP) [151]. Phosphodiesterase inhibitors (PDEis) are small molecules that interfere with the conversion of cyclic nucleotides into noncyclic forms propelled by PDE[152]. PDEis modulates neuroinflammation, inhibits microglial activation, and antagonizes pro-inflammatory cytokine production (Table 2) [153-155]. Studies have confirmed that several selective PDEis, including inhibitors of PDE1, PDE4, PDE5, PDE7, and PDE10, restore cAMP and cGMP levels and reverse the expression of inflammatory genes in LPS-treated microglia [153-158].

Combining cilostazol or sildenafil (PDE3i and PDE5i, respectively) with ATT decreased the bacterial burden in M. tb-infected lungs and shortened the time to lung sterilization in mice [159, 160]. Similarly, PDE4i roflumilast, FDA-approved therapy for severe chronic obstructive pulmonary disease (COPD), reduces IL-1β levels and obtains better clearance than ATT alone in the chronic TB mouse model after 8 weeks posttreatment [161]. In contrast, rolipram and cilomilast, also belonging to type 4 PDEi, are detrimental and they shorten the time to death in M. tb-infected mice [160]. An investigational new drug, PDE4i CC-11050, an experimental novel medication with increased stability compared to CC-3052, has been evaluated for safety and efficacy in several clinical trials. It was reported to alleviate inflammation-related tissue damage while improving bacterial burden and clinical outcomes in isoniazid-treated rabbits with pulmonary TB [162, 163]. A phase II open-label human clinical trial using CC-11050 as an adjunct treatment is currently being conducted in South African patients with TB (NCT02968927). There is plenty of research on the application of cilostazol and other types of PDEis in the integrity of the BBB, prevention of brain edema, and other supports of brain function; therefore, focusing on the potential of PDEis in TBM is promising [164].

## Statins

Statins (3-hydroxy-3-methyl-glutaryl-CoA reductase inhibitors) are widely used to treat dyslipidemia. Recent studies have also indicated their roles in neuroinflammation, phagosome maturation, and autophagy (Table 2) [165, 166]. They modulate the function of various immune cells, including microglia, T cells, neutrophils, and NK cells. In addition, statins partially inhibit microglial activation and reduce neuronal apoptosis through controlling chemokines and cytokines such as CCL5 and CXCL10, IL-10, and TGF-β1 [167, 168]. They also impair phagocytosis by affecting the microglial secretion of nitric oxide (NO), TNF-α, IL-1β, and brain-derived neurotrophic factor (BDNF). Moreover, they reduce the expression of surface antigens, including major histocompatibility complex II (MHC II) and chemokine receptors CCR5 and CXCR3 [169-171]. Because microglia/macrophages contain abundant lipids, statins can interrupt cholesterol metabolism, attenuating the favorable cellular environment for M. tb growth and persistence in the brain [172, 173]. A meta-analysis of 2,073,968 patients suggested that statin application for treatment decreases the risk of TB infection by approximately 40% in the general population [174]. Similarly, other studies have discovered that statins have greater anti-infective efficacy in patients without diabetes [175, 176]. In an *in vitro* study, infected macrophages from atorvastatin-treated patients showed a significant reduction in M. tb burden compared with those from healthy controls. When treated with simvastatin or rosuvastatin, intracellular bacterial growth in macrophages was limited in a mouse model in which lung burdens and histopathological abnormalities were improved [177]. The combination of statins (pravastatin, simvastatin, and fluvastatin) with ATT has shown to be a potential therapeutic application against intracellular M. tb in mice. For example, pravastatin modulates phagosome acidification and the proteolytic activity of macrophages [178]. An ongoing phase II clinical trial by Johns Hopkins University assesses whether pravastatin adjunctive therapy for TB will shorten the mean time to sputum culture conversion and improve lung function outcomes compared to the conventional regimen (NCT03456102). Considering the similarities between macrophages and microglia, it is essential to consider the therapeutic effects of statins in the treatment of TBM.

## TNF-α Antagonists

During various infectious conditions, TNF-α is a pro-inflammatory cytokine crucial in initiating and promoting inflammation, especially in forming granulomas [179, 180]. In TBM, mainly from microglia, TNF-α activates immune response, which contributes to the recruitment of peripheral immune cells like macrophages and neutrophils after a pathogenic encounter [181, 182]. TNF-α restricts the cerebral bacilli replication and dissemination in CNS during the early phase of M. tb infection, allowing the innate immune response to remain activated and persistent for several weeks [182]. However, abnormal TNF-α secretion may be harmful to neurons, as described in neuropathological contexts like PD, AD, ischemia, and infectious diseases such as Zika virus-induced encephalitis, and particularly in TBM, where hyperinflammation, caseous necrosis, and cachexia are all correlated with elevated TNF-α [183-187].

*In vivo* rabbit models infected with the recombinant strain of M. bovis bacillus Calmette-Guérin (BCG) Montreal, which expresses the murine gene for TNF-α, show a heavy burden of mycobacterial colony forming units in the brains, severe meningeal inflammation with thickening of the leptomeninges and large numbers of mononuclear cells. These changes indicate that high expression of TNF-α may contribute to neuroinflammation and leaks in the BBB [185]. Therefore, modulating TNF-α expression may be a potential therapeutic strategy, and two clinical trials are currently ongoing (Table 2).

TNF-α inhibitors like infliximab and etanercept are effective in treating Crohn’s disease and rheumatoid arthritis (RA) [188, 189]. Numerous clinical cases have reported the use of infliximab therapy after ATT, which effectively mitigated PR in CNS-TB [85, 190]. Combining etanercept with standard TB treatment in mice resulted in a significantly lower pulmonary bacterial burden and relapse rate than those of the standard TB treatment [139, 191]; another study reported similar results [192]. In a phase I study, etanercept showed favorable responses and was safe for TB treatment in HIV-1 positive groups [193]. Conversely, TNF-α inhibitors increase the risk of latent TB or new infection in patients with RA, inflammatory bowel disease, or other inflammatory diseases. The statistic shows an approximately 4-fold increase in TB in those treated with TNF-α antagonists compared to those not treated with it [194, 195]. The median time to TB onset after initiating TNF-α antagonist treatment varies depending on the type of inhibitor used. Infliximab and adalimumab have a median time of 3-6 months, whereas etanercept requires more than 1 year [196]; the *in vivo* investigations may provide insights into these phenomena. When anti-TNF-α antibody (e.g., infliximab) or TNF-neutralizing TNF receptor fusion molecule (e.g., etanercept), a blocker for soluble TNF-α, were administered to infected mice, TNF-α bioactivity was neutralized equivalently. Both treatments worsened the disease in the acute phase of M. tb infection, and the mice became moribund. Notwithstanding, the anti-TNF-α antibody aggravated bacterial burden in the established infection, impairing granuloma organization in injected mouse lung sections. In contrast, control and mTNFR2-Fc-treated (as modeled directly on human etanercept) mice maintained a lower bacterial burden and proper granuloma structures. The disparity in the detection of these reagents may be due to low mTNFR2-Fc penetration into the granuloma or immune complex formation of TNF and anti-TNF-α antibodies [197].

Meanwhile, intervention with different types of TNF-α also leads to different immune responses. Soluble TNF (sTNF) is involved in chronic inflammatory diseases, whereas transmembrane TNF (tmTNF) maintains innate immune functions. Using a dominant-negative mutant of TNF (DN-TNF) to block sTNF while excluding tmTNF attenuates arthritis without suppressing innate immunity to *Listeria monocytogenes* [198]. DN-TNF protects against acute liver inflammation induced by an endotoxin challenge in M. bovis BCG-infected mice, whereas etanercept compromises host immunity against acute M. bovis BCG and M. tb infections [199]. A recent study developed a novel vaccine (DTNF114-TNF114) that targets TNF epitopes 1-14 and partially controls the activity of tmTNF and its receptor. In the mCherry-BCG mouse model, DTNF114-TNF114 reduced sTNF levels without suppressing the host immune response [200]. Therefore, it is crucial to understand the roles of sTNF and tmTNF when using anti-TNF agents as complementary strategies for antibiotic treatment. Vaccines or agents that specifically inhibit excessive sTNF expression while leaving tmTNF signaling alone are promising strategies to reduce immunological damage and complications induced by hyperinflammation while inhibiting M. tb.

Unfortunately, agents like etanercept and infliximab have poor BBB penetration, limiting their use in TBM treatment [201]. In some TBM case reports, adjunctive usage of thalidomide, a TNF-α protein synthesis inhibitor capable of crossing the BBB, benefits in controlling neuroinflammation during antibiotic treatment [202-204]. In these cases, thalidomide was introduced as an immunomodulatory agent several months after poor response to antibiotics and corticosteroids. In a clinical trial involving 15 children with TBM, adjunctive thalidomide therapy appeared to be safe and well tolerated, with remarkable clinical outcomes compared to patients receiving standard treatment for TBM [205]. It also remarkably decreased the level of TNF-α in plasma and CSF. However, the same research group terminated the clinical trial on a larger scale, in which thalidomide caused adverse effects and death when doses as high as 24 mg/kg/day were administered orally [206]. Nevertheless, 37 of 38 children receiving adjunctive thalidomide therapy (3-5 mg/kg/day) showed satisfactory clinical and radiological responses without apparent side effects in a retrospective cohort study of over 10 years in South Africa [207]. Currently, thalidomide cannot be routinely used to treat TBM; however, it may be useful as a prophylactic “salvage therapy” in patients who are resistant to antibiotic drugs or high-dose corticosteroids. Although thalidomide analogs, including lenalidomide and pomalidomide, are more effective than thalidomide in inhibiting TNF-α produced by microglia, their therapeutic effects on TBM have received less attention [201, 208]. The sole study that combined the application of a thalidomide analog with ATT in a rabbit model of TBM demonstrated that the thalidomide analog reduced morbidity and mortality while considerably improving survival and was more effective than thalidomide alone [209].

## Vitamin D

Vitamin D, whose biologically active form is 1,25-dihydroxyvitamin D3, exerts immunomodulatory effects on microglia/macrophages via vitamin D receptors. The functions of Vitamin D in autophagy and innate immunity have been extensively studied [210, 211]. Vitamin D levels in children with TB or latent TB are significantly lower, and vitamin D deficiency is particularly associated with TBM and pulmonary TB cases [212, 213]. Nursing home residents with severe vitamin D deficiency (defined as 25-hydroxyvitamin D levels < 10 ng/mL) have a higher risk of developing active TB than the general population [214]. The immunological effects of vitamin D against M. tb in macrophages are associated with innate immune activation and phagolysosomal activity. Notably, adding active vitamin D restores the M. tb disruption of phagosome maturation [215]. TLRs, as discussed, play a role in the recognition and internalization of M. tb, and the activation of TLRs upregulates the expression of Vitamin D receptors in macrophages/monocytes. Moreover, TLR activation triggers the downstream gene cathelicidin, known as LL-37, a member of the cathelicidin family of antimicrobial proteins, which is abundant in macrophages, monocytes, and neutrophils during M. tb infection and is upregulated in activated microglia [216-218]. The LL-37 pathway is indispensable for activating the transcription of the autophagy-related genes *Beclin-1* and *Atg5* and the colocalization of mycobacterial phagosomes with autophagosomes in human macrophages [219]. Although Vitamin D promotes an anti-inflammatory state in microglia, thereby limiting the microglial cell polarization process, there is no direct evidence of the therapeutic effects of vitamin D in TBM [220, 221]. Current studies indicate that vitamin D deficiency might attenuate immunity against M. tb (Table 2); however, more meticulous studies are required to provide conclusive data for additional vitamin D supplementation, resulting in superior resistance to TB infection [222].

## Discussion

**Figure 2.**
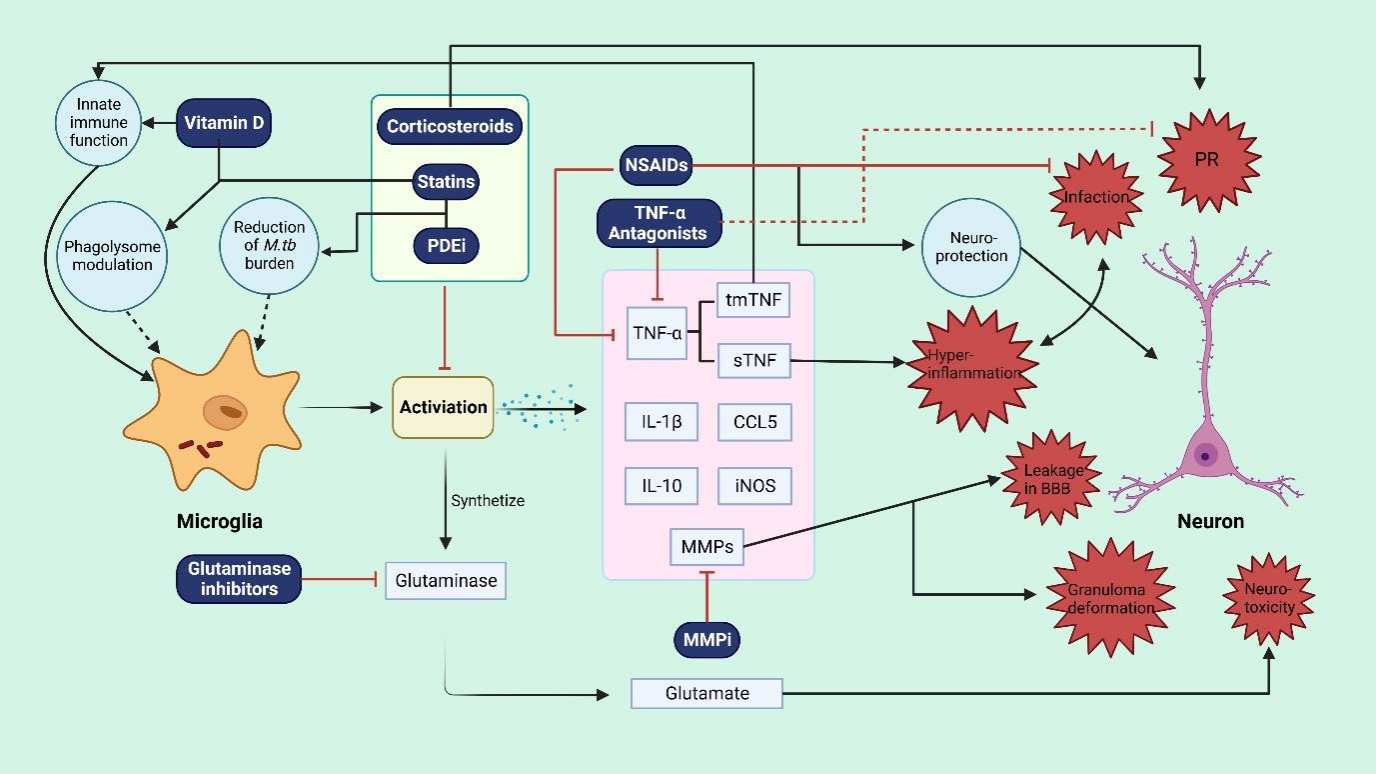
**Overview of the emerging HDT targets for microglia during M**. tb infection. Once activated by the recognition of M. tb, microglia function as immune surveillance agents to remove bacilli. The drugs listed in this figure target various pathways to interrupt inflammatory reactions. The dashed lines indicate the need for more direct evidence to prove an absolute correlation between symbols. The figure was created with Bio-Render. com.

The presence of the BBB that shapes the unique immunological environment in the CNS restricts the treatment of TBM to aspects other than the sites of TB. The ideal approach for treating TBM entails effectively eliminating M. tb with good pharmacological penetration and limiting inflammatory injury and complications caused by abnormal immune activation. Microglia, immune surveillance cells centered in the loop between glial cells and neurons, are promising targets, and HDT can maintain a reasonable balance between pro- and anti-inflammatory responses. This review summarizes eight categories of promising HDT drugs that target microglia for TBM treatment (Fig. 2).

Several traditional or newly developed drugs associated with HDT with different classifications and inflammatory modulation mechanisms for treating TBM are frequently tested in various clinical trials. In light of the previously discussed therapies, we highlighted several HDTs from different classes that are steadily progressing as adjunctive methods to ATT. However, HDT has several disadvantages. First, they exhibit off-target effects. For instance, corticosteroids and NSAIDs are widely used broad-spectrum inflammatory inhibitors. Although it is recommended that dexamethasone or prednisolone can be used to treat TBM, especially in cases where hydrocephalus and other neurological deficits develop, corticosteroids may exhibit an intricate effect based on leukotriene A4 hydrolase (LTA4H) genotype polymorphisms, in which the hyper-inflammatory genotype receives more benefits from corticosteroids than other genotypes; the hypo-inflammatory genotype predicts an inadequate intracerebral inflammatory response with lower CSF cytokine concentrations than those in the hyper-inflammatory group, associated with death from TBM [2, 223]. A randomized, double-blind clinical trial (NCT03100786) is currently being conducted to verify the efficacy of adjunctive dexamethasone in HIV-negative patients with TBM, stratified by LTA4H genotype [224]. Compared to corticosteroids, NSAIDs are more likely to prevent new brain infarctions without causing irreversible microglia damage, which leads to efficient microglial phagocytosis. However, one study has shown that traditional NSAIDs are associated with an increased risk of active TB [225]. Similarly, some PDEis, including rolipram and cilomilast, may accelerate the progression.

Selective antagonists, including drugs, small-molecule compounds, and antibodies against specific cytokines/chemokines, can be game-changers in restraining M. tb infection and immune injury appropriately without negatively damaging microglial function. Moreover, as TNF-α is also pivotal in maintaining granuloma structure, selective inhibition of different types of TNF-α could lead to a discrepancy in the outcome. Hence, a second challenge was established on the path to the application. Because some HDTs have poor BBB penetration and severe side effects, the delivery system, drug stability in the brain, and side effects must be thoroughly tested. Despite its anti-TNF-α effects and well BBB penetration, thalidomide is notorious for its historical teratogenicity and modulation of T cell subsets [226]. These facts continue to limit it as a “salvage therapy” even if there are positive results in clinical trials. Similarly, new glutaminase inhibitors exhibit poor storage stability and toxicity and thus require more investigation. The application of non-invasive dynamic positron emission tomography (PET) could be useful for discovering intralesional drug distributions and related pharmacokinetic properties in the brain. Statistics show that children with TBM benefit from a high dose of rifampicin, and those with higher rifampicin distribution in the CSF have fewer neurological sequelae [227, 228]. Meanwhile, the use of ^11^C-rifampin provides similar results. PET imaging revealed that rifampin penetration into brain lesions is limited, and pharmacokinetic modeling predicted that a high dose of rifampin (30 mg/kg) is required for optimal intralesional drug distribution for treating pediatric patients with TBM [229]. In addition to dose adjustments, PET offers an attractive opportunity to explore novel anti-TB drugs [230]. Hence, more experimental methods, including PET, should be developed in future studies.

Finally, some of the fundamental mechanisms are required to be elucidated. Internalization of M. tb by microglia occurs partially via unidentified PRRs that differ from those found in macrophages. The participation of PRRs in microglial phagocytic capacity is tightly associated, and downstream cellular signaling pathways determine the evasion of tubercle bacilli. Although microglia are not the only glial cells that secrete pro-inflammatory factors in response to M. tb, they may serve as an initial and persistent source and then collaborate with other cell types, including astrocytes and oligodendrocytes, to shape the miscommunicated milieu during TBM. Many HDT drugs act on multiple cell types. Therefore, it is necessary to determine the interactions between cells during drug treatment. Further studies are required to confirm the neuronal excitotoxicity of glutamate, which has recently become prominent in TBM.

Due to the current state of research, the studies and clinical trials in this review are derived largely from the research in macrophages and pulmonary TB. As TBM is the most severe complication of TB, applying existing pulmonary TB treatments to TBM is critical. Therefore, we recommend that more attention should be paid to the precise design and thorough execution of TBM-related clinical trials. Adjunctive HDTs are not well-known as a ‘one-size-fits-all’ approach, and detailed knowledge of the relationship between microglia and neuro-inflammation would provide better treatment tailored to patients.
